# Editorial: Deep learning for multimodal brain data processing and analysis

**DOI:** 10.3389/fnins.2023.1264015

**Published:** 2023-08-18

**Authors:** Liansheng Wang, Lequan Yu, Baptiste Magnier

**Affiliations:** ^1^Department of Computer Science, Xiamen University, Xiamen, China; ^2^Department of Statistics and Actuarial Science, The University of Hong Kong, Pokfulam, Hong Kong SAR, China; ^3^Euromov Digital Health in Motion, Mines-Telecom Institute Alès, Alès, France

**Keywords:** deep learning, multimodal, brain data, medical image analysis, brain image analysis

The processing and analysis of brain data are of great importance for understanding the workings of the human brain and diagnosing neurological disorders, brain tumors, and more. Deep learning can process complex relationships in brain data both automatically and effectively, demonstrating strong generalization capabilities. In recent years, it has gradually emerged as a common method for addressing complex medical problems related to the brain.

Deep learning-based methods offer a comprehensive, accurate, and fast approach to processing and analyzing brain data. An impressive achievement is that Gong et al. proposed a high-performance multi-task framework with both regression and classification capabilities, which solved the problem of slow efficiency in evaluating IntraCerebral Hematoma (ICH) volume using Non-Contrast head Computed Tomography (NCCT). This method even achieved evaluation results comparable to those of clinical doctors in the task of assessing ICH hematoma volume. Using deep learning for brain segmentation can improve the accuracy of brain partition volume prediction. Using predictions of brain partition volume, Pan et al. explored the correlation between 5-min cognitive test (FCT) scores used for cognitive impairment screening and brain structure. To enhance the ability of deep learning models to segment East Asian brains, Moon et al. reported a high-performance brain segmentation algorithm based on a 3D convolutional neural network for East Asian brain segmentation.

By applying deep learning to multimodal brain data processing and analysis, we can fully harness the potential of multimodal data. This approach combines different types of brain data for a more comprehensive and accurate analysis. To help prevent stroke in patients with carotid atherosclerosis, Lv et al. trained a multimodal model with a channel attention mechanism using multimodal brain MRI imaging data to predict stroke diagnosis incidence. The channel attention mechanism can learn significant regions in different modal images, effectively improving the classification performance of the model.

There are three notable issues to consider in this Research Topic.

Firstly, the interpretability of medical deep learning models is of paramount importance. Gong et al. addressed this in the Research Topic by introducing a method known as Gradient-weighted Class Activation Mapping (Grad-CAM). This technique can generate heat maps with high interpretability, enabling the localization of brain hemorrhage sites.

Secondly, the integration of brain data processing results with other disciplines can lead to new discoveries in brain science. For instance, Pan et al. identified a pronounced correlation between 5-min Cognitive Test (FCT) scores and volumes of hippocampal-related regions and the amygdala in populations with cognitive impairments.

Lastly, model bias is a significant concern when utilizing pre-trained deep learning models. While many deep learning techniques have found successful application in brain data processing and analysis, a large number of pre-trained models are trained on Caucasian brain data, which can result in bias. To address this, Moon et al. trained a deep learning model using an extensive dataset of East Asian brain MRI scans, creating a model specifically calibrated for this ethnic group.

Advanced deep learning methods hold the potential to extract meaningful information from multimodal brain data. The collected research in this topic, “*Frontiers in deep learning for multimodal brain data processing and analysis*”, can significantly contribute to the field of brain medical research.

## Author contributions

LW: Writing—original draft, Writing—review and editing. LY: Writing—original draft, Writing—review and editing. BM: Writing—original draft, Writing—review and editing.

